# Supplementary material for: Polygenic architecture of flowering time and its relationship with local environments in the grass Brachypodium distachyon
Source: Genetics. 2024 Mar 20;227(1):iyae042. doi: 10.1093/genetics/iyae042 (PMC11075549; doi:10.1093/genetics/iyae042)
Supplement: iyae042_Supplementary_Data [file iyae042_supplementary_data.zip › Supplemental_Figures_GENETICS-2024-306789.pdf]

Supplementary Figures

Polygenic architecture of flowering time and its relationship with local environments in the grass *B. distachyon.*

Nikolaos Minadakis, Lars Kaderli, Robert Horvath, Wenbo Xu, Michael Thieme, Daniel D. Woods and Anne C. Roulin

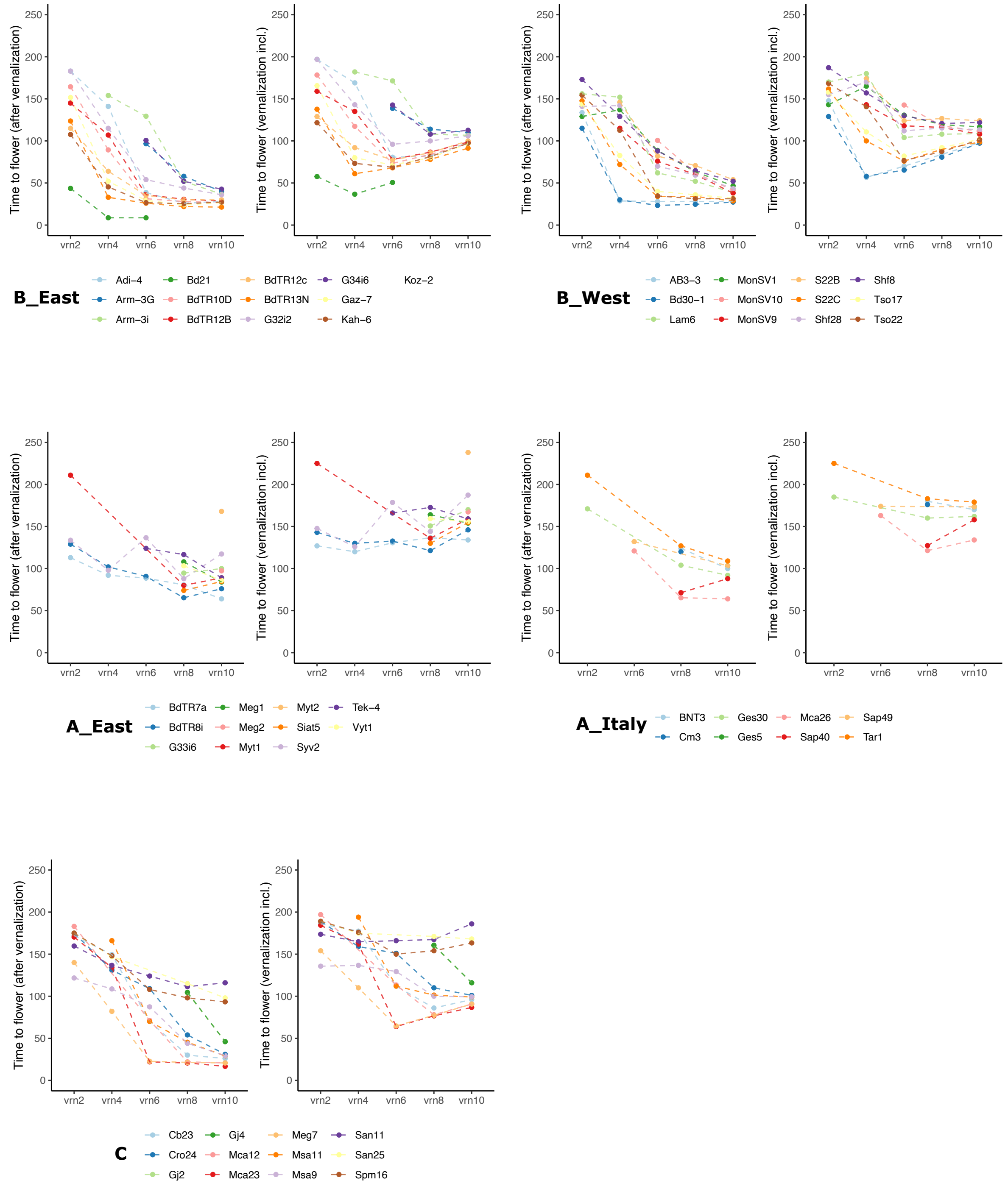

Fig. S1: Flowering time results**.** For each genetic clade, plots display the number of days given accessions (mean over replicates) take to flower after or including vernalization time.

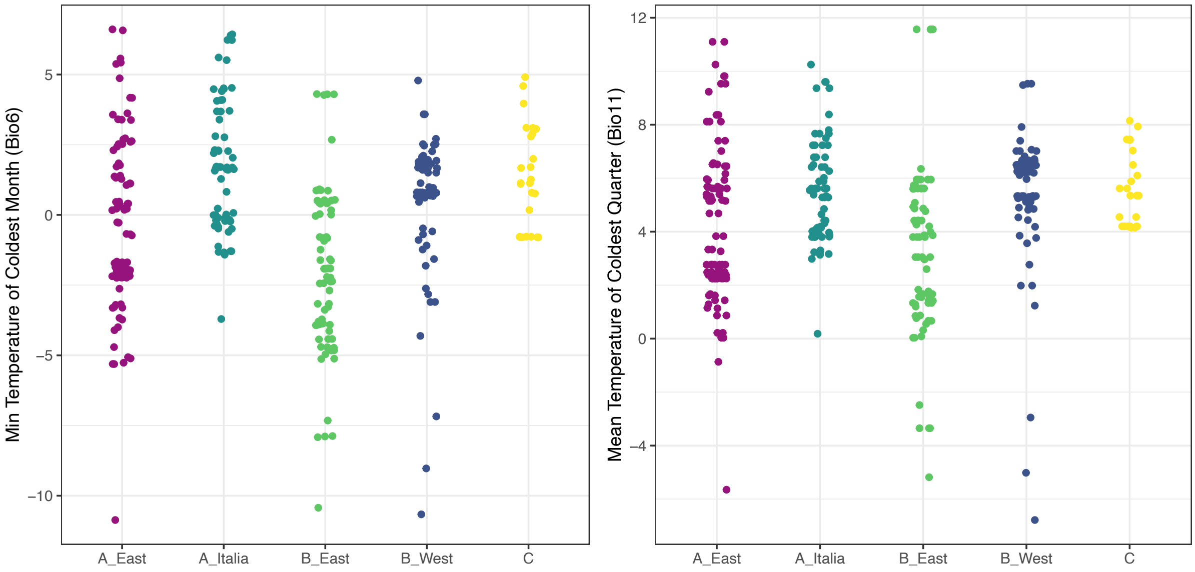

Fig. S2: Bio6 and Bio11 distribution across the 332 accessions of the diversity panel.

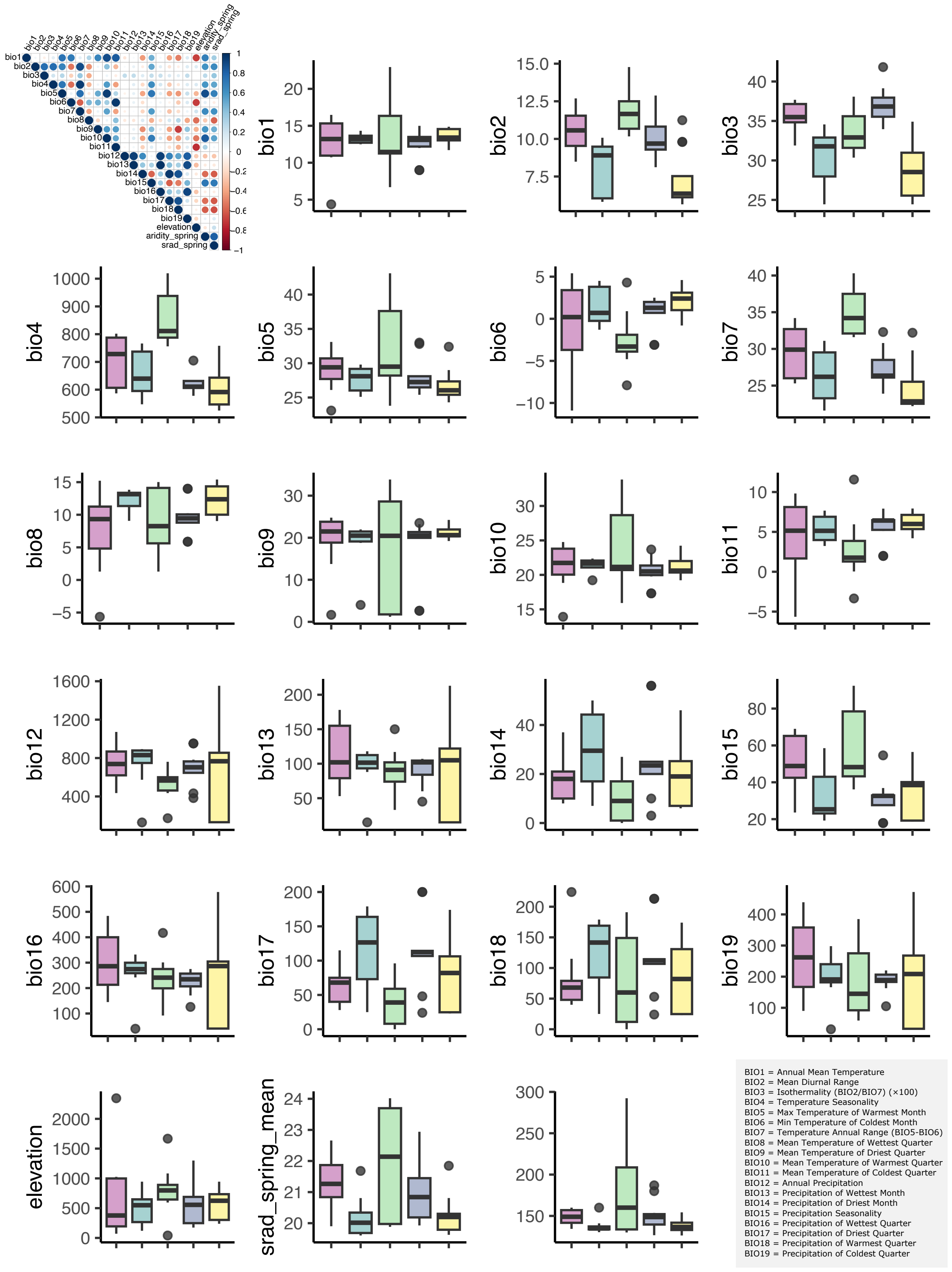

Fig. S3: Bioclimatic variable variation among the five genetic clades and 56 accessions chosen for the greenhouse experiment. The correlogram display levels of correlation among variables.

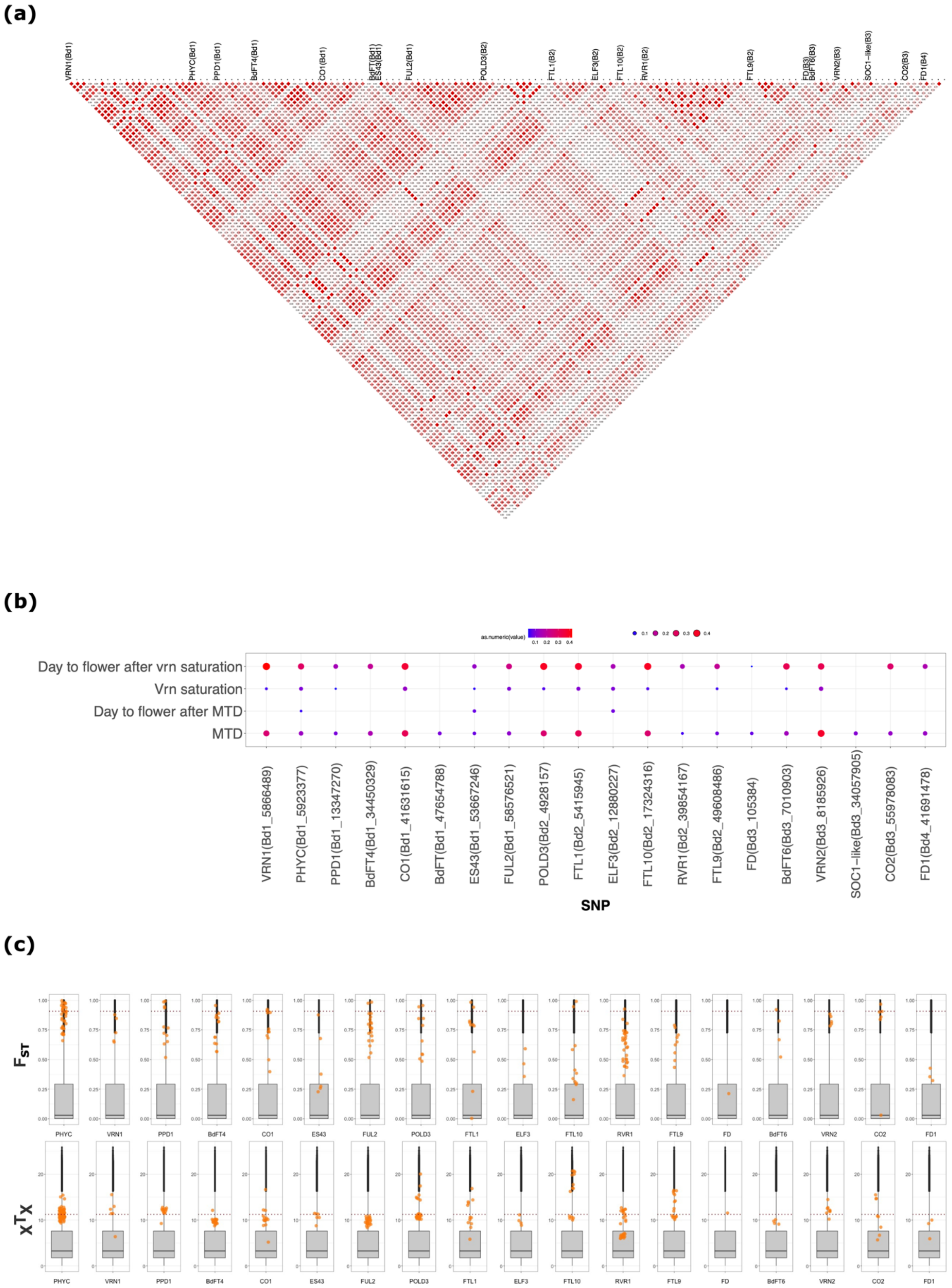

Fig. S4: Flowering time genes**.** (a) Linkage disequilibrium across the 20 flowering time genes associated with one of the flowering time-related traits (143 SNPs). The annotated SNPs indicate the first SNP of each gene (b) RDA output for the 20 top SNPs associated flowering time-related traits. (c) F_{ST} and X^TX statistics computed in the entire diversity panel.

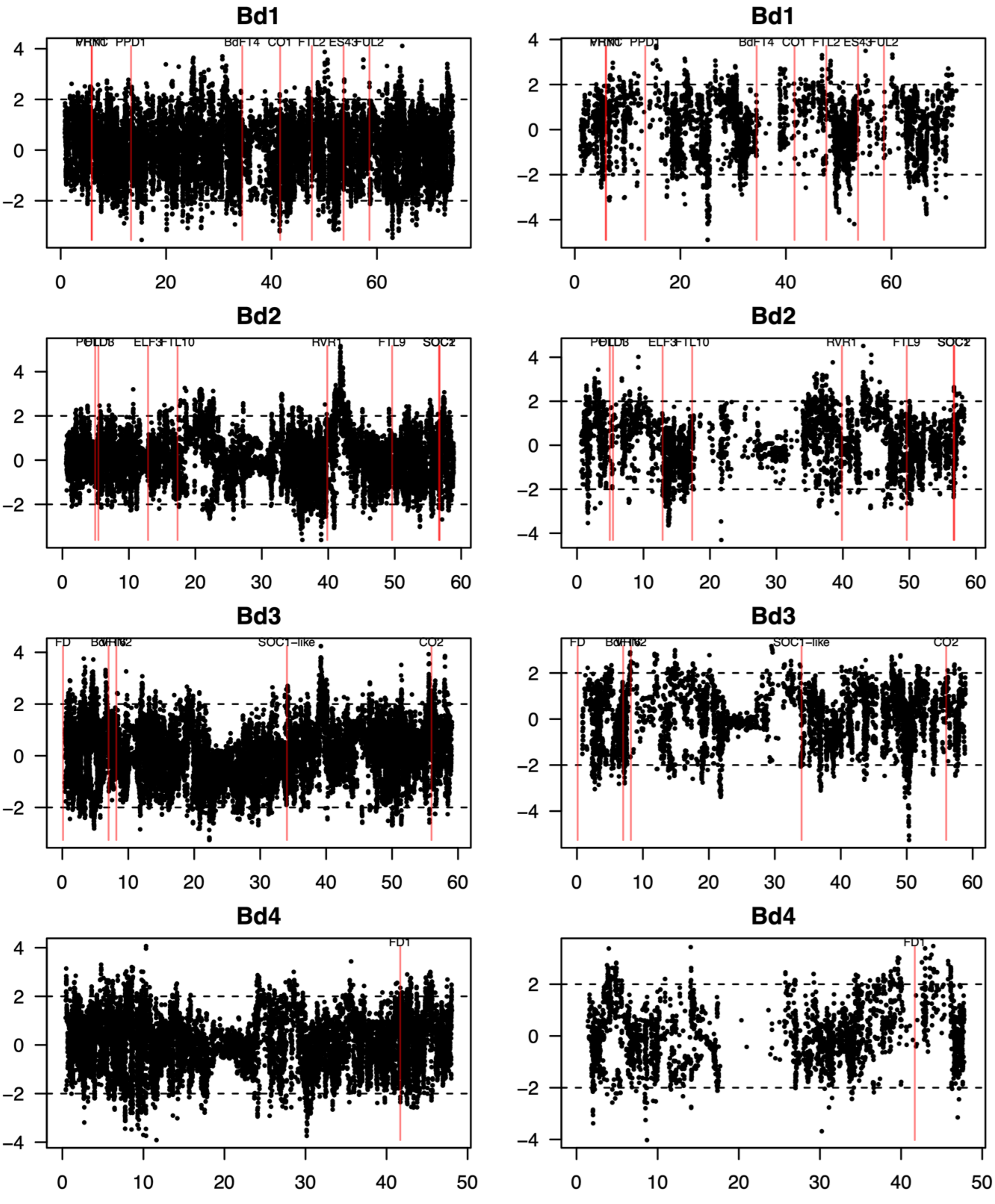

Fig. S5: integrated Haplotype Score (his) computed in the A (left panels) and B (right panely) lineages respectively.

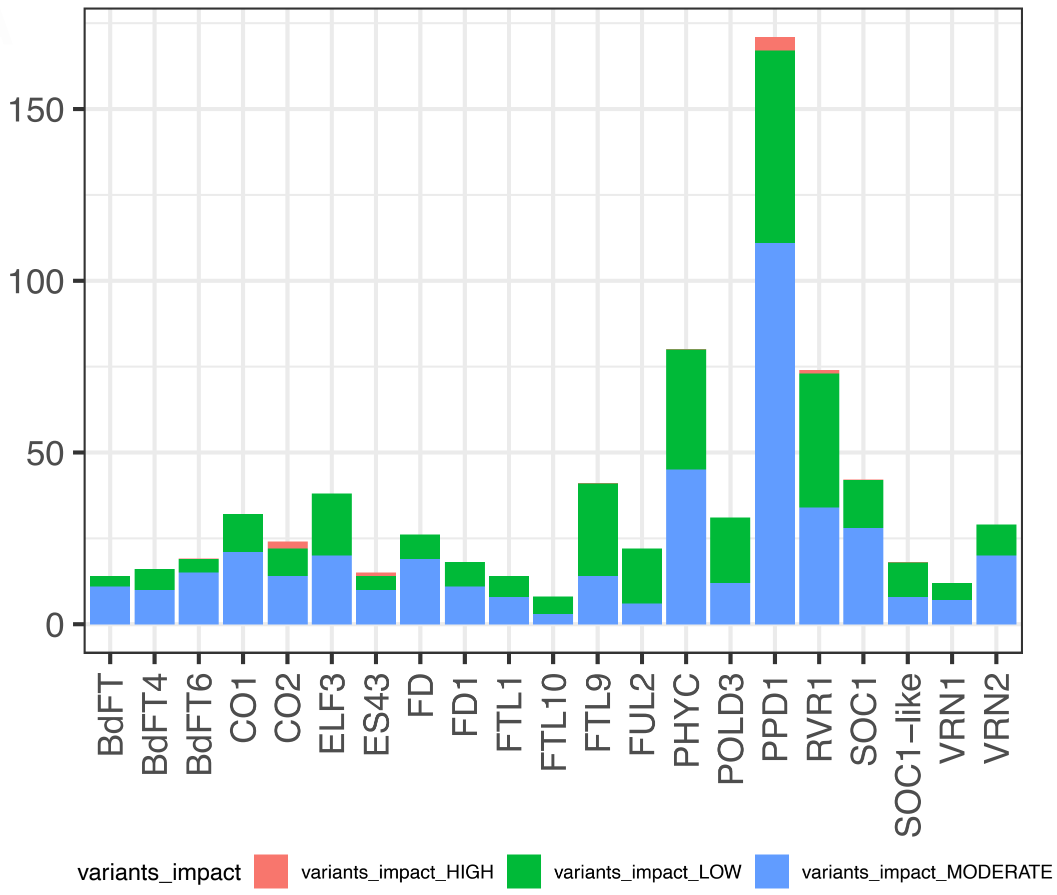

Fig. S6: SnpEff output for the flowering-time genes

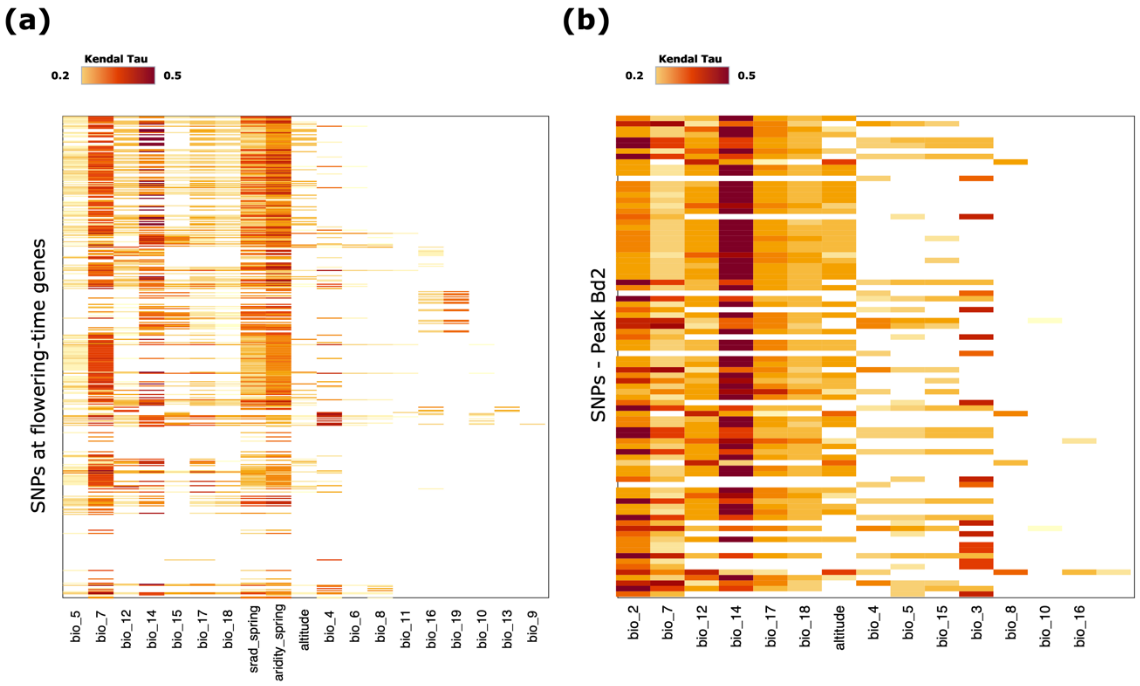

Fig. S7: Kendall correlation between SNP at AFT-genes and bioclimatic variables a) Heatmap displaying the association between SNPs at SFT-genes and bioclimatic variables. Variables not significantly associated with SNPs are not displayed b) Most significantly associated SNPs in flowering-time genes showing a significant association with at least one bioclimatic variable c) Heatmap displaying the association between SNPs at GWAs peaks (outdoor experiment). Variables not significantly associated with SNPs are not displayed

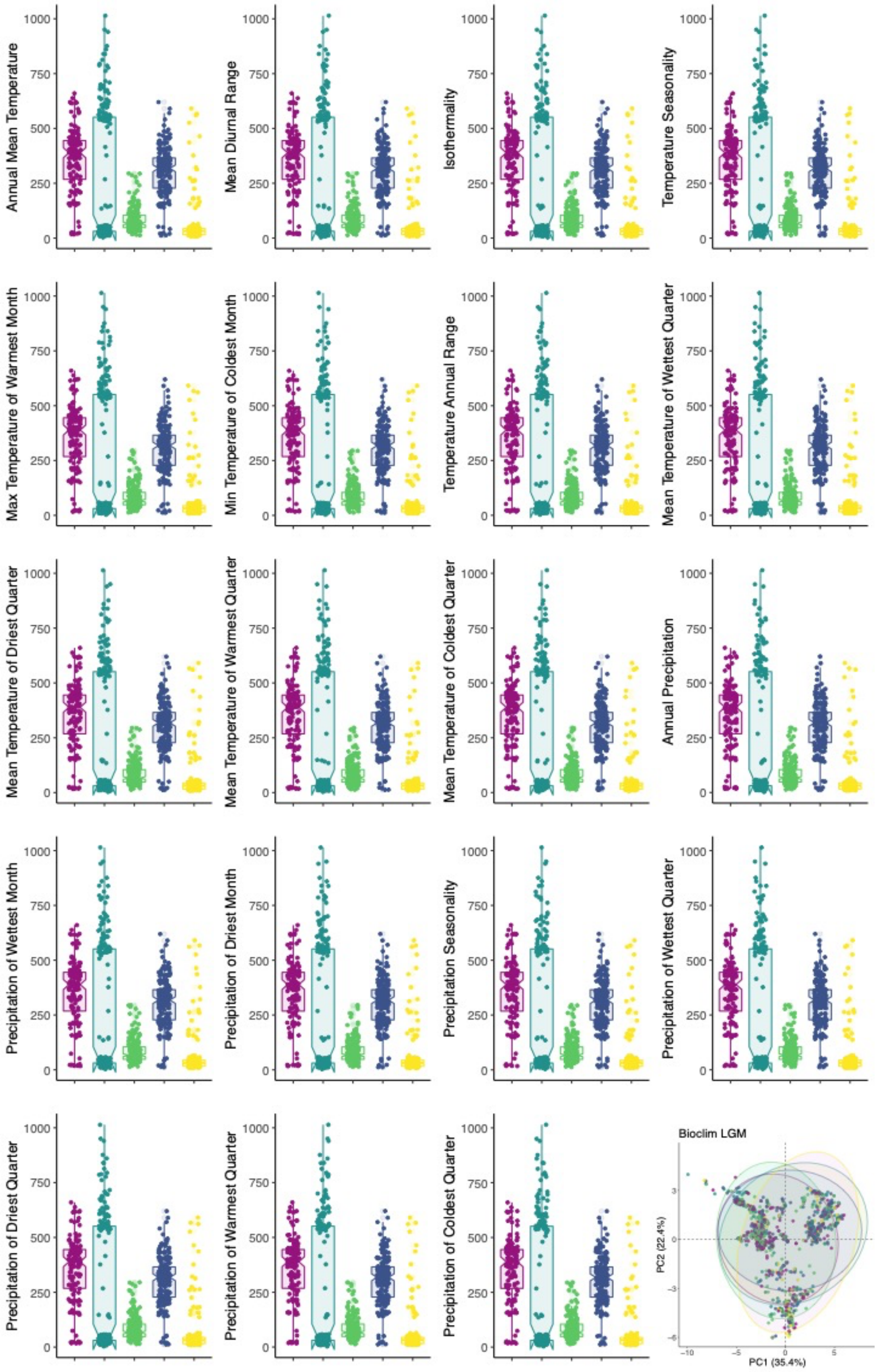

Fig. S8: Bioclimatic variables distribution over the five genetic clades during LGM. The PCA was performed using the 19 bioclimatic variables displayed by the boxplots.

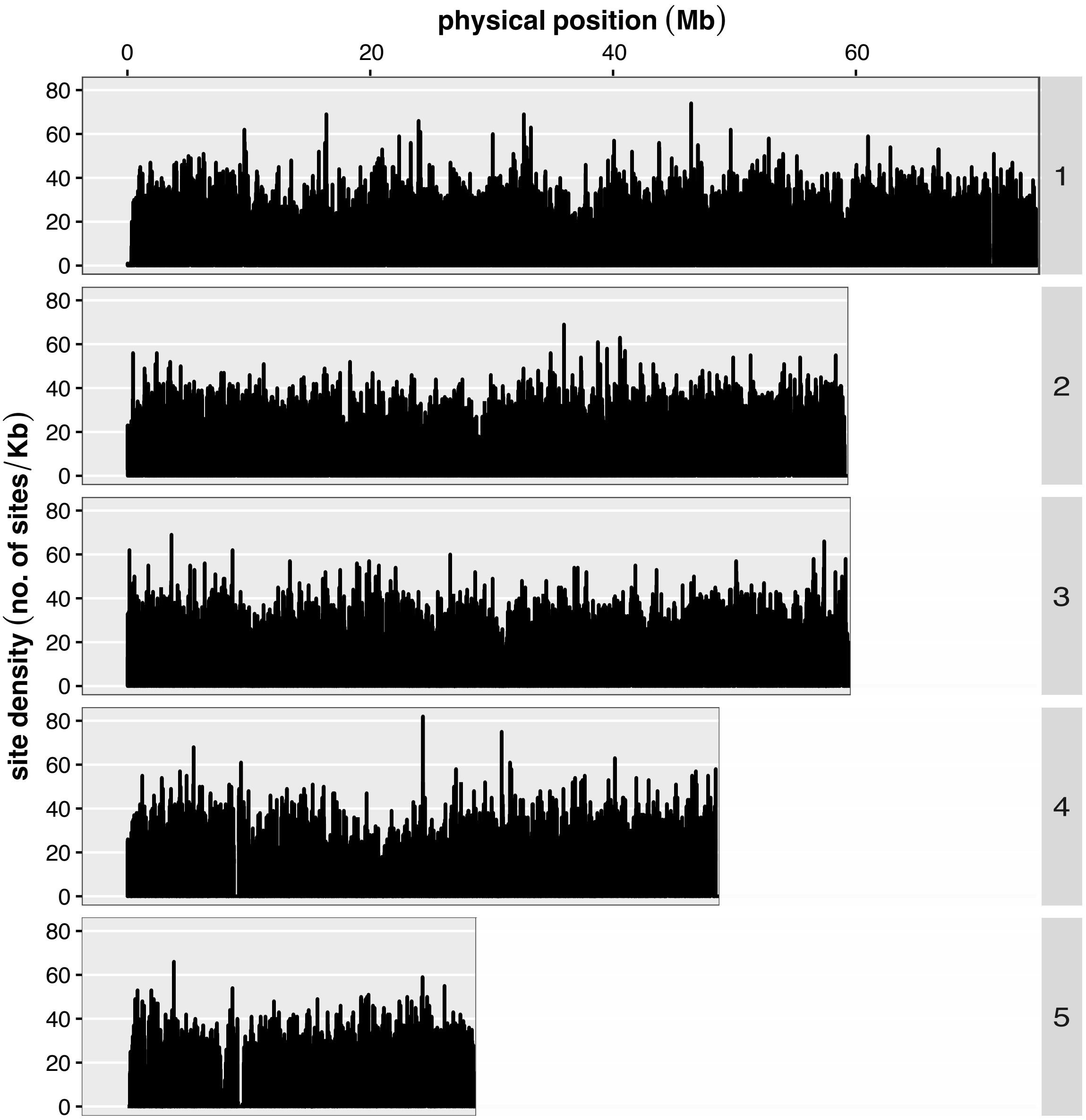

Fig. S9: Marker density along the five chromosomes. The plot displays the 2,266,225 SNPs kept for the GWAs
